# Enhanced Mechanical Properties of Metallic Glass Thin Films via Modification of Structural Heterogeneity

**DOI:** 10.3390/ma14040999

**Published:** 2021-02-20

**Authors:** Xindi Ma, Kang Sun, Peiyou Li, Nizhen Zhang, Qing Wang, Gang Wang

**Affiliations:** 1 Institute of Materials, Shanghai University, Shanghai 200444, China; xindima@shu.edu.cn (X.M.); dadani@shu.edu.cn (N.Z.); qingwang@shu.edu.cn (Q.W.); 2 School of Materials Science and Engineering, Shaanxi University of Technology, Hanzhong 723001, China; lipeiyou112@163.com

**Keywords:** metallic glass thin films, magnetron sputtering, structure, nanoindentation, nano-scratch

## Abstract

The structure of Cu_50_Zr_50_ and Co_56_Ta_35_B_9_ metallic glass thin films (MGTF) was effectively tailored via various applied substrate temperatures by means of the magnetron sputtering technology. Obviously enhanced hardness and elastic modulus are achieved by different compositional MGTFs by increasing the substrate temperature. Compared with the CuZr MGTFs, the CoTaB MGTF deposited at 473 K displays the smaller strain-rate sensitivity exponent, *m*, and a weaker spectrum intensity based on the nanoindentation creep test, suggesting its better creep resistance. In addition, the STZ volume of the CoTaB MGTF significantly decreases after depositing at higher temperature. According to the nano-scratch analysis, the CoTaB MGTF at the substrate temperatures of 473 K performs the shallower scratch width and the larger *H*^3^/*E*^2^ value, indicating its better tribological property.

## 1. Introduction

Metallic glass thin film (MGTF), as a kind of two-dimensional metallic glass, usually exhibits some excellent properties that are well-known, such as higher strength and toughness, larger elastic limit, better corrosion and wear resistances owing to its amorphous structure, as compared with conventional crystalline metal film [1,2]. The reported MGTFs with the focus of understanding of liquids and glasses, provide model systems for studying some long-standing fundamental issues, and have potential engineering and functional applications [3].

The application of bulk metallic glasses (BMGs) is hindered by a lack of macroscopic ductility at room temperature (RT) [4]. The deformation mechanisms of BMGs have been explained in several theories or models, such as free volume theory [5], the shear-transformation zone (STZ) model [6], and efficient cluster packing theory [7,8]. Recent work reveals that BMGs have intrinsically structural heterogeneities that are usually termed as plastic units or heterogeneous “defects” [9]. These structural heterogeneities at nanoscale [8,10] can be homogenized by annealing [11]. In this case, the strength and thermal stability of BMGs can be improved by annealing due to an annihilation of heterogeneities. So far, the relationship between the plastic units and the macroscopically mechanical properties of BMGs is still lacking.

The development of the super-stable glass, which is a kind of glassy film with excellent thermodynamic, dynamic, and mechanical properties [12,13], enlightens us to quickly homogenize the glassy phase based on the fast dynamics of surface atoms in the glassy film. To manipulate the dynamics of surface atoms in the film, the environmental temperature during the process of vapor depositing glassy film, i.e., the substrate temperature, *T*_sub_, is a key factor. The mobility of atoms or molecules on the surface significantly increases with increasing the *T*_sub_ value [14], which allows the atoms to easily jump to stable configurations [15]. Thus, the structure of glassy films can be changed by changing the *T*_sub_ value without the significant modification of the glassy structure [16,17,18]. As such, the modification of the *T*_sub_ value during the process of vapor deposition is an effective method to investigate the influence of structural heterogeneity on the macroscopic properties.

In this paper, a Co_56_Ta_35_B_9_ (at.%) MGTF with a high glass-transition temperature, *T*_g_, and a Cu_50_Zr_50_ MGTF with a low *T*_g_ are selected as model materials. The Cu_50_Zr_50_ alloy has a glass forming ability (GFA) in the binary Zr-based metallic glass system [19], and the Co_56_Ta_35_B_9_ alloy performs high hardness and better wear resistance [20,21]. Based on the vapor-deposition technique, the glassy structures of the MGTFs are effectively modified by changing the *T*_sub_ value. The effect of different *T*_sub_ values on the glassy structure is discussed, and the mechanical properties of various MGTFs is systematically analyzed. Our work provides more experimental evidences for the structural heterogeneity-design, which is of benefit to the application of the MGTF.

## 2. Experimental Procedure

A direct-current planar magnetron sputtering device (JPG-450, Shenyang ZKY Technology Development Co., Shenyang, China) was sued to deposited the Cu_50_Zr_50_ and Co_56_Ta_35_B_9_ MGTFs in a high-vacuum chamber (5 × 10^−5^ Pa) with a load-lock system, and a probe handler. The targets were compound with a nominal composition of Cu_50_Zr_50_ (at.%) and Co_56_Ta_35_B_9_ (at.%). The deposition substrates were a monocrystalline silicon wafer and a glass wafer with dimensions of 10 × 10 × 0.5 mm^3^. The distance between the substrate and the target was 80 mm, and the MGTFs were deposited for 2 hours. Before the deposition, pre-sputtering was carried out for 30 minutes. The operated power and pressure were 60 W and 0.7 Pa, respectively, in a high purity argon atmosphere. The substrate was continuously rotating to guarantee the homogeneity during the deposition. The deposition temperature, i.e., the *T*_sub_ value, was set at room temperature, RT, (without intentional heating), and 473 K for each film. As revealed by previous works, the crystallization usually occurred during annealing near 0.8 *T*_g_ [22,23]. The glass transition temperature, *T*_g_, of Cu_50_Zr_50_ is 670 K, which is lower than that of Co_56_Ta_35_B_9_ (961 K). Considering the heat generation phenomenon during sputtering, a deposited temperature of 473 K is within the reasonable range for maintaining the glassy structure of Cu_50_Zr_50_. Simultaneously, in order to investigate the structural changes induced by the composition at the same deposited temperature, 473 K is also applied for Co_56_Ta_35_B_9_. The film thickness range was 2 μm by sputtering for 2 h. The microstructure of the MGTFs were examined by an X-ray diffractometer (XRD) (18 KW D/MAX2500, Rigaku Company, Tokyo, Japan) with a Cu-Kα radiation of 30 kV.

Nano-scratching experiments were conducted in a TI-900 TriboIndenter machine (TI-950, Hysitron, USA) with a Berkovich tip. The tip radius is 150 nm and a half-angle is 65.35°. The initial tip-shape calibration was tested using a fused silica standard sample. The scratch length was 200 µm, and the moving speed of the indenter was 2 µm/s. Nano-scratch testing was conducted at a loading force of 1 mN. At this loading force, at least two scratches were repeatedly performed. Nanoindentation tests were performed with a diamond Berkovich tip mounted on a TriboScope nanomechanical testing system (TI-950, Hysitron, Minneapolis, MN, USA). The nanoindentation measurements were performed under load-control mode at RT. The system was fitted with a Berkovich indenter. In order to obtain the accurate nanoindentation data, the indenter was cleaned by pure aluminium and calibrated by fused silica before testing. The tests were operated in a load-control mode with a maximum load of 8 mN. The maximum indentation depth of 8 mN was controlled at around 10% of the film thickness so that to avoid the interference from the substrate [24]. We also adopted an 8 mN holding test to examine the machine stability and the influence of thermal drift on the experimental result. To ensure the credibility of measurement results, the holding times for the creep and hardness measurements were 40 and 5 s, respectively. The loading time of 40 s was chosen to test creep behavior, and the loading time of 5 s was chosen to directly obtain modulus and hardness. From the nanoindentation loading–unloading curve, hardness, *H*, and Young’s modulus, *E*, were calculated according to the Oliver and Pharr approach [19].

## 3. Results

**Figure 1:**
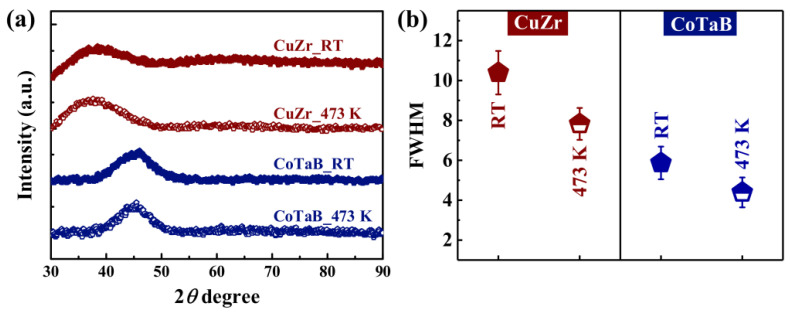
Structural analysis of the CuZr and CoTaB MGTFs deposited at RT and 437 K. (**a**) XRD patterns. (**b**) The full width of half maximum, FWHM, as a function of various MGTFs.

Figure 1a shows XRD patterns of the CuZr and CoTaB MGTFs prepared at different deposition temperatures, i.e., *T*_sub_. Only one broad diffuse peak without no apparent crystalline phase is displayed, indicating the microstructure of four films are fully glassy. The first peak can be well fitted by pseudo-Voigt function as shown in Figure S1, which gives the values of the peak position, *q*, and the full width of half maximum (FWHM). The *q* value of various MGTFs can be seen in the Supplementary Materials. Figure 1b shows the FWHM as a function of various treated MGTFs. the CuZr and CoTaB MGTFs display the same tendency with increasing the substrate temperature. The FWHM value of the CuZr MGTFs decreases from 10.39 ± 1.09 to 7.83 ± 0.80, while for the CoTaB MGTFs, it changes from 5.87 ± 0.82 to 4.39 ± 0.75 corresponding to the films treated at RT and 473 K. The larger FWHM value indicates a more disordered structure in the glass. Therefore, with increasing the substrate temperature, the structure of the film prepared at higher temperature (473 K) is more ordered, which is found in both compositions.

**Figure 2:**
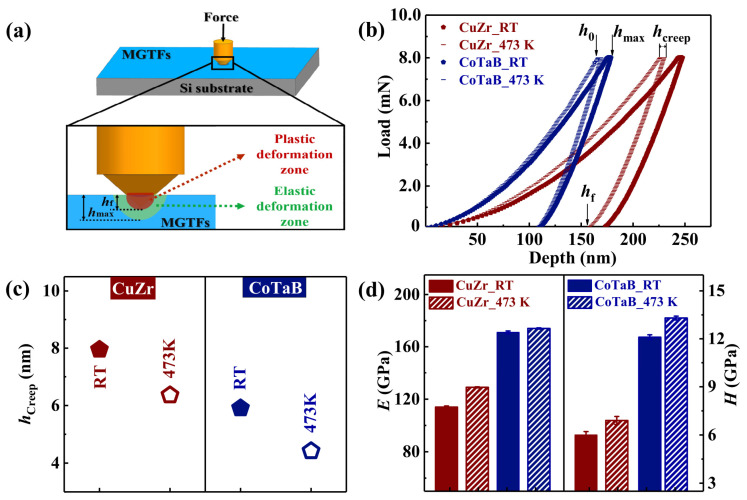
The analysis of nanoindentation tests of the CuZr and CoTaB MGTFs deposited at RT and 437 K. (**a**) The schematic diagram of the nanoindentation measurement. (**b**) The typical load–displacement curves. (**c**) The creep depth, *h*_creep_, as a function of various MGTFs. (**d**) The values of the elastic modulus, *E*, and the hardness, *H* as functions of various MGTFs.

Figure 2a shows the schematic diagram of the nanoindentation process. The MGTF is deposited on the silicon wafer, and the following creep measurement was performed on the surface of the film. As displayed, when the load is applied, a plastic and an elastic deformation zones underneath the indenter will accordingly appear. As shown in Figure 2b, the representative load vs. displacement curves for four MGTFs are presented. Since the applied loading rate is fast, the pop-in event is suppressed in the *P*-*h* curves, which is in agreement with the previous work [25,26,27]. The characteristic depth, e.g., the initial and the maximum displacement of the creep stage, *h*_0_, and *h*_max_, the creep depth, *h*_creep,_ = *h*_max_ − *h*_0_, and the final deformation depth, *h*_f_, are significantly different of various MGTFs. Their corresponding values are listed in Table 1. Compared to the MGTFs deposited at RT, the values of *h*_0_, and *h*_max_, for the MGTFs treated at 473 K are decreased, both for the CuZr and CoTaB MGTFs. The creep depth, *h*_creep_, as a function of various MGTFs is plotted in Figure 2c, displaying the same tendency for the CuZr and CoTaB MGTFs with increasing the substrate temperature from RT to 473 K. The *h*_creep_ values of the CuZr MGTFs prepared at RT and 473 K are 7.95 ± 0.37 nm and 6.36 ± 0.49 nm, respectively. For the CoTaB MGTFs, the values of *h*_creep_ at RT and 473 K are 5.91 ± 0.25 nm and 4.42 ± 0.34, respectively. Therefore, the higher substrate temperature treated films with a more ordered structure exhibits a smaller creep depth. In addition, compared with the different compositions, the creep depth of CoTaB MGTFs is smaller than that of CuZr MGTFs not only at RT but also at 473 K. The final deformation depth, which is the residual indentation depth reflecting the degree of plastic deformation in the indentation process, corresponding to the plastic deformation zone displayed in Figure 2a. It shows the smaller value for the MGTFs deposited at 473 K, indicating a smaller area of the plastic deformation zone in the more ordered MGTF during the nanoindentation process. The deviation between *h*_max_ and *h*_f_ reflects the elastic recovery after the indentation process, which represents the elastic deformation zone shown in the schematic diagram. The values of (*h*_max_ − *h*_f_) of the CuZr MGTFs are larger than those of the CoTaB MGTFs. With increasing substrate temperatures from RT to 473 K, its value is slightly decreased. The values of the elastic modulus, *E* and the hardness, *H*, of various MGTFs are shown in Figure 2d. The *E* and *H* values of the CuZr MGTFs deposited at RT are 114.4 ± 0.5 GPa and 6.1 ± 0.2 GPa, respectively. When the substrate temperature increases to 473 K, the *E* and *H* values of the CuZr MGTFs increases to 129.0 ± 0.4 GPa and 6.9 ± 0.3 GPa, respectively. For the CoTaB MGTFs deposited at RT, the *E* and *H* values are 171.1 ± 1.1 GPa and 12.1 ± 0.2 GPa. The increased deposition temperature leads to the larger *E* and *H* values, which are 174.3 ± 0.5 GPa and 13.3 ± 0.1 GPa, respectively. The *E* and *H* values of two glassy films increase with increasing the *T*_sub_ value from RT to 473 K. In addition, their corresponding values of the CuZr MGTFs are lower than those of the CoTaB MGTFs. The comparison of the modulus (*E*) and the hardness (*H*) of two metallic glasses measured by nanoindentation are listed in Table 2. Compared to the previously reported results, the values of CuZr MGTF calculated in our work are within the range of error. The values of CoTaB MGTF are lower than those of bulk metallic glass. Since there were no reported values of CoTaB MGTF from other works, more works should be done to give a comprehensive comparation.

**Figure 3:**
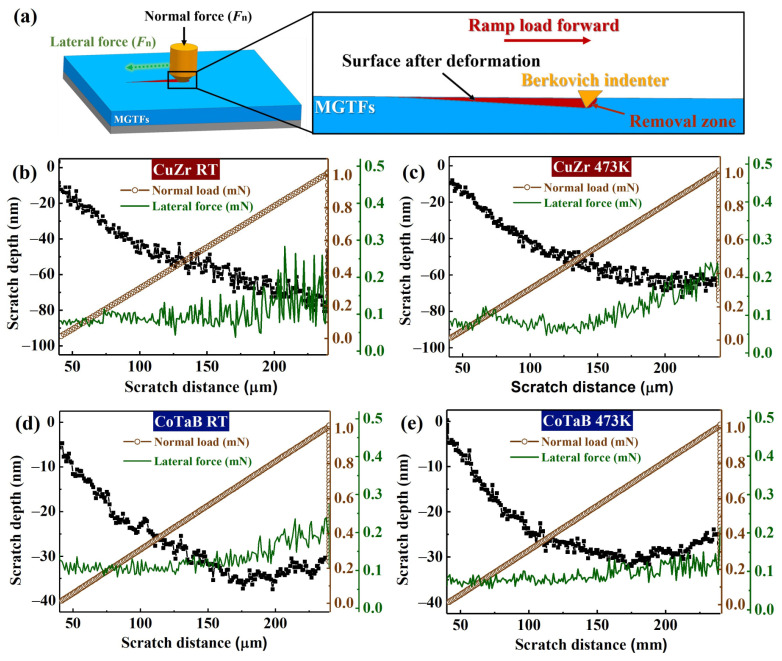
The analysis of nano-scratch tests of CuZr and CoTaB MGTFs treated at RT and 437 K. (**a**) The schematic diagram of the nano-scratch process. (**b**) The scratch depth, the normal load, the lateral force as a function of the CuZr MGTF deposited at RT. (**c**) The scratch depth, the normal load, the lateral force as a function of the CuZr MGTF deposited at 473 K. (**d**) The scratch depth, the normal load, the lateral force as a function of the CoTaB MGTF deposited at RT. (**e**) The scratch depth, the normal load, the lateral force as a function of the CoTaB MGTF deposited at 473 K.

Figure 3a provides the profile of the nano-scratch process with an applied ramping normal load mode, where a normal force and a lateral force were performed on the MGTFs’ surface. As shown in Figure 3b, a ramp of the normal force from 0 to 1.05 mN within 100 s is applied on the CuZr MGTF at the *T*_sub_ of RT, it causes a lateral force to increase from approximately 0.08 mN to 0.25 mN with a force fluctuation that exhibits a maximum fluctuation magnitude of 0.2 mN. With ramping the normal force, the scratch depth linearly increases from ~ 40 to ~ 95 μm. For other three MGTFs, the nano-scratch processes are summarized and plotted in Figure 3c–e. It is obvious that the lateral force and the scratch depth for other three MGTFs exhibit the same evolution trend as the case in the CuZr MGTF at RT.

## 4. Discussion

In the glassy phase, the homogeneously atomic structure means that the activation of heterogeneous defects is difficult due to their higher energetic stability and the large energy absorption capability [27]. In the elastic stage, an elastic-static stress can induce a small amount of “structural disorder”, which, in turn, leads to the creation of excess free volume, i.e., the heterogeneous defects increase. Thus, it is required to explore the heterogeneous-defect evolution. A creep test is carried out.

**Figure 4:**
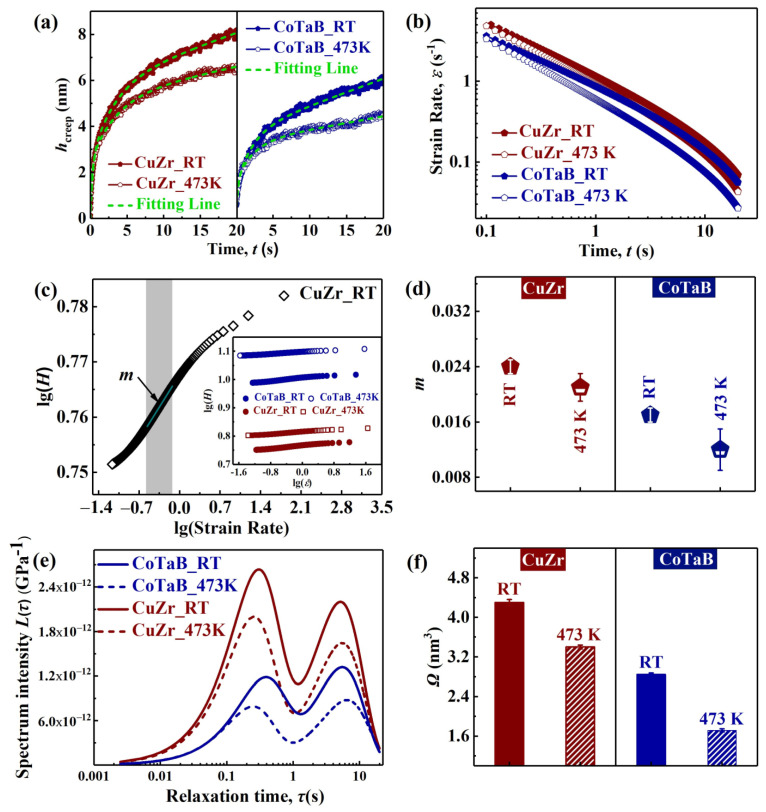
The analysis of the creep measurements of the CuZr and CoTaB MGTFs deposited at RT and 437 K. (**a**) The creep depth as a function of the time. The dotted line represents the fitting curves calculated by Equation (1). (**b**) The creep strain rate as a function of the time. (**c**) Logarithm (*H*—curves. The strain-rate sensitivity exponent, *m*, obtained from fitting the liner part of the curve. The inset image is the Logarithm (*H*—$\dot{\varepsilon }$) curves for four MGTFs. (**d**) The strain-rate sensitivity exponent, *m*, as a function of various MGTFs. (**e**) The relaxation spectrum of various MGTFs. (**f**) The volume of the STZ, Ω, as a function of various MGTFs.

According to the nanoindentation creep measurement, the structural responses of the MGTFs can be deduced via the compliance spectrum and retardation spectrum of creep. The displacement during indentation hold includes the instantaneous elastic deformation, plastic deformation, viscoelastic deformation, and viscous flow [28]. The viscoelastic including an anelastic deformation and a viscoplastic deformation can be described as a series of linear springs and dashpots, known as the Kelvin-Voigt model [28,29,30]:

(1) $$h=\sum_{i=1}^{n}h_{i}\left(1-\left(1-e^{-t/\tau_{i}}\right)\right)+t/\mu_{0}$$

where *h_i_* is the indentation depth, *τ_i_* is the characteristic relaxation time for the activation of the *i*-th anelastic process, *t* is the experimental time, and *μ*_0_ is a constant proportional to the viscosity coefficient of the last dashpot. The creep curves of four MGTFs are shown in Figure 4a, where the light-blue dash line indicates the fitting curves by Equation (1). The corresponding values of the fitting parameters are listed in Table 3. The values of *h*_1_ and *h*_2_ decrease with increasing the substrate temperature from RT to 473 K both for the CuZr and CoTaB MGTFs. With regard to the first relaxation time, *τ*_1_, it increases with increasing the substrate temperature. However, for the second relaxation time, *τ*_2_, it decreases when the treated temperature from RT to 473 K. At the same deposited temperature, the indentation depth, *h_i_*, and the characteristic relaxation time, *τ_i_*, values of the CuZr MGTFs are larger than those of the CoTaB MGTFs. Moreover, the *μ*_0_ value of the CuZr and CoTaB MGTFs increases with increasing substrate temperature. A higher *μ*_0_ value indicates a progressively raised viscosity with structural relaxation [30], being associated with the localized reduction of free volume. Therefore, the concentration of free volume decreases with increasing the substrate temperature, resulting in the larger hardness shown in Figure 2d.

The creep strain-rate, $\dot{\varepsilon }$, during the holding stage can be calculated as Equation (2):

(2) $$\dot{\varepsilon }=\frac{dh}{dt}\frac{1}{h}$$

Accordingly, the strain rate as a function of creep time is plotted in Figure 4b. It is evident that the strain rates of four MGTFs decrease from 5 s^−1^ to 3 × 10^−2^·s^−1^ with creep time. The strain rate of the MGTFs deposited on the substrate at RT is always higher than the case at 473 K. With regard to the composition, the strain rate of the CuZr MGTFs are always higher than that of the CoTaB MGTFs. In order to further characterize, the strain-rate sensitivity exponent, *m*, is calculated. The *m* value is measured from the slope of the logarithm (*H* − $\dot{\varepsilon }$) curve, i.e., $m=\frac{\partial \mathrm{lg}\left(H\right)}{\partial \mathrm{lg}\left(\dot{\varepsilon }\right)}$, which is plotted in Figure 4c. Based on the fitting curves, the *m* values are estimated. As shown in Figure 4d, the *m* values of CuZr MGTFs deposited on RT and 473K substrate are 0.024 ± 0.001 and 0.021 ± 0.002, respectively. For the CoTaB MGTFs, they are 0.017 ± 0.001 and 0.012 ± 0.003 corresponding to the films deposited on RT and 473 K. It is obvious that the high substrate temperature causes that a low strain-rate sensitivity, *m*, in the MGTFs. A quite low *m* value in MGs is generally attributed to a strongly localized shear flow, which suggests a good creep resistance of the MGTF [30]. Therefore, the CoTaB MGTFs with the substrate temperature of 473 K performs the better creep resistance than other experimental films. Moreover, in the macroscopic scale, the creep properties are correlated with the *E* and *H* values of the MGTFs. The CuZr and CoTaB MGTFs deposited at 473 K exhibits better creep resistance.

The anelastic component of the creep can be analyzed in terms of a spectrum of relaxation times. The isothermal relaxation spectra can be calculated using the following approximated expression [4,30]:

(3) $$L\left(\tau \right)=\frac{A_{0}}{P_{0}h_{in}}{\left[\sum \left(1+\frac{t}{\tau_{1}}\right)\frac{h_{1}}{\tau_{1}}e^{-t/\tau_{1}}+\sum \left(1+\frac{t}{\tau_{2}}\right)\frac{h_{2}}{\tau_{2}}e^{-t/\tau_{2}}\right]}_{t=2\tau }$$

where *A*_0_*/P*_0_ is the inverse of the hardness of *H*, and *h_in_* is the maximum indentation depth. Figure 4e shows the retardation spectrum of the four MGTFs according to Equation (3), which consists of two peaks with two well-defined relaxation times. These two peaks, i.e., two relaxation processes, represent two kinds of different deformation units. With increasing the substrate temperature from RT to 473 K, the intensities of two peaks decrease, which suggest that the relaxation intensities of two relaxation processes decrease. For the CuZr MGTF, the intensity of the first relaxation process decreases about 24.15%, and the second relaxation decreases about 25.28% due to the increases of the substrate temperature. It can be seen the decreases of the intensities for two relaxation processes of the CuZr MGTF is almost same. For the peak positions, i.e., the relaxation times for the relaxation processes, the increase in the substrate temperature reduces the first relaxation time about 15.59%, i.e., the peak position shifts to the small value, and improves the second relaxation time about 5.91% for the CuZr MGTF. However, for the CoTaB MGTF, the changes in the intensity and the times of two relaxation processes are larger than the cases of the CuZr MGTF with the substrate temperature from RT to 473 K. The intensities of the first relaxation and the second relaxation processes decrease approximately 66.80% and 33.44%, respectively, in the CoTaB MGTF. The first relaxation time reduces about 43.96%, and the second relation time increases about 18.19%, respectively.

The reduction in the peak intensity indicates a decrease in the population of the corresponding defects. The shift of the peak position to the short relaxation time can be attributed to a decreased difficulty of the activation of the remaining defects [30]. The peak intensity of the CoTaB MGTF is significantly weaker than that of the CuZr MGTF, which indicates that the quantity of the heterogeneous defects of the CoTaB MGTF is smaller than that of the CuZr MGTF. When the alloy is homogeneous, the cooperative motion of shear flows is relatively easy [31]. However, as some heterogeneous short-range ordered structures existed in the alloy, the distribution of free volume will change, and the localized shear flows will be blocked.

The STZ volume can be accordingly estimated by the cooperative shear model (CSM) of Johnson and Samwer [7], based on the obtained *m* values from creep. In the CSM model, the volume of the STZ, Ω, is defined as [32]:

(4) $$\Omega =\frac{0.018\times \sqrt{3}KT}{mHR\zeta r_{c}^{2}\left(1-\frac{\tau }{\tau_{c}}\right)}$$

where *T* is the temperature, *k* is the Boltzmann constant, *m* is strain-rate sensitivity exponent, constants *R* ≈ 1/4, *ζ* is a constant (ζ ≈ 3), *γ*_C_ is the critical shear strain (*γ*_C_ = 0.027), *τ*_C_ is the critical shear strength, and *τ*/*τ*_C_ = 0.711 [32]. Figure 4f depicts the variation tendency of the estimated STZ volume as a function of various MGTFs. As it shown, from RT to 473 K, the STZ volume of the CuZr MGTFs are 4.30 ± 0.06 and 3.40 ± 0.04, respectively, which are higher than the CoTaB MGTFs of 2.80 ± 0.03 and 1.70 ± 0.03 at same deposition environment. With the same composition, the volume of the STZ decreases with increasing of the substrate temperature to 473 K. The variation of the CuZr MGTFs and the CoTaB MGTFs is 20.9% and 39.3%, respectively, suggesting the structure of the CoTaB MGTF is strongly influenced by the substrate temperature. It may be attributed to the different ratio between *T*_sub_, and the glass transformation temperature, *T*_g_, i.e., *T*_sub_/*T*_g_, which are 0.706 and 0.492 of the CuZr and CoTaB MGTFs, respectively [20,23]. Further work should be done to explore the structure evolution. The inhomogeneous structure of the MGs will be characterized by nanoindentation, nano-dynamic mechanical analyzer (nano-DMA) and dynamic atomic force microscopy, as well as high resolution transmission electron microscopy (HR-TEM).

**Figure 5:**
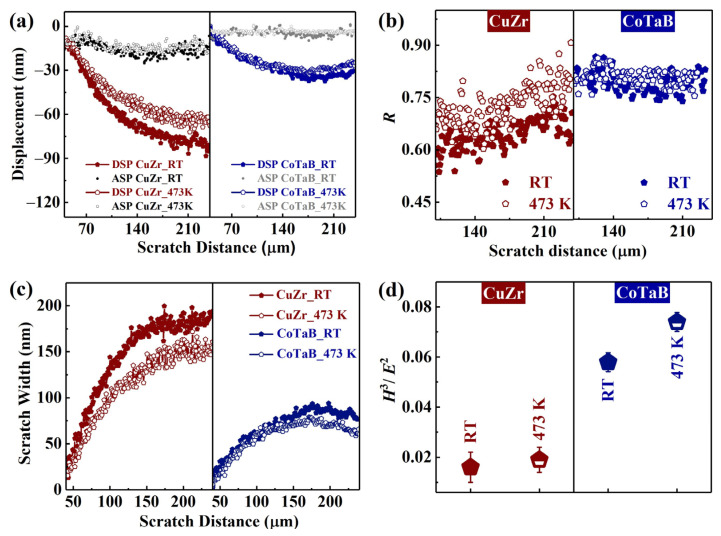
The tribological analysis of the CuZr and CoTaB MGTFs deposited at RT and 437 K. (**a**) The penetration depth, *d_P_*, and the residual depth, *d_R_*, as functions of scratch distances. (**b**) The ratio, *R*, $R=\left(d_{P}-d_{R}\right)/d_{P}$, as a function of scratch distances. *d_P_* represents the penetration depth during the nano-scratching process, and *d_R_* represents the residual depth after the nano-scratching process. (**c**) The scratch width as a function of scratch distances. (**d**) The value of *H*^3^/*E*^2^ as a function of various MGTFs.

The glassy phase under the indenter is subjected to the plastic and elastic deformation not only in the indentation process but also in the scratching process [33]. The elastic deformation under the indenter leads to the decrease in the residual scratch depth, i.e., *d_R_*. [33]. Figure 5a shows the penetration depth during the nano-scratching process, *d_P_*, and the residual depth after the nano-scratching process, *d_R_*, as functions of various MGTFs. It can be seen that the *d_P_* and the *d_R_* values of the CuZr MGTFs deposited at RT and 473 K are always larger than those of the CoTaB MGTFs. The indentation depth of the MGTFs prepared at RT is deeper than that of the films prepared at 473 K. Meanwhile, compared to CoTaB MGTF, the CuZr MGTF is more easily affected by *T*_sub_. With regard to the same composition, the *d_P_* values of the films deposited on the substrate at RT are larger than those deposited at 473 K. However, the *d_R_* of the film deposited at 473 K is similar to those at RT. In order to quantitatively characterize, The ratio, *R*, is introduced, which is defined as $R=\left(d_{P}-d_{R}\right)/d_{P}$, reflecting the degree of elastic recovery of the nano-scratch test. Figure 5b displays the *R* value as a function of the scratch distance after 100 μm. With increasing the scratch distance, the alteration of *R* displays obvious positive correlation for the CuZr MGTFs deposited at RT and 473 K. However, the value of *R* for the CoTaB MGTFs is basically unchanged. The larger elastic recovery indicates the more energy the material can absorb, which results in the less quantity of cracks, suggesting a better wear resistance of materials [34]. Therefore, the CoTaB MGTFs with larger *R* values performs better tribological property.

The wear resistance of materials can be also characterized by means of the scratch width. In general, a better wear resistance will result in the shallower scratch width. During the nano-scratch process, the scratch width, *W*, can be calculated as following [35]:

(5) $$W=2\sqrt{3}dtan\theta$$

where *d* is the indentation depth, θ is an intrinsic geometric parameter of indenter (θ = 65.3°). Figure 5c shows that the scratch width as a function of the scratch distances. As shown, the increased substrate temperature leads to the reduction of *W*. Moreover, the scratch width of the CoTaB MGTFs is significantly smaller than that of the CuZr MGTFs, indicating the better tribological property.

During the scratch progress, the lateral force, *F*_l_, can be calculated as [35]:

(6) Fl=24.5[hmax−(FmaxH2.263E2)12]2τ

It is strong correlated with the hardness and the modulus, where *h_max_* is the maximum depth, *F_max_* is the maximum load, *E* is the elastic modulus, and *τ* is the shear strength. As shown in Figure 3b–e, the reduced *F*_l_ suggests the better wear resistance. As revealed by previous works, when a rigid sphere with a radius of *r* is pressed into an elastic/plastic half-space, the load, *P*_y_, which requires to initiate plastic deformation estimated as [36,37,38]:

(7) $$P_{y}=0.78r^{2}\frac{H^{3}}{E^{2}}$$

The *H*^3^/*E*^2^ values of four MGTFs are shown in Figure 5d. The *H*^3^/*E*^2^ values of the CuZr MGTFs shift from 0.016 to 0.019 with increasing the *T*_sub_ from RT to 473 K. With regard to the CoTaB MGTFs deposed on RT and 473 K, they are 0.061 and 0.078, respectively. It indicates the requirement to initiate plastic deformation for CoTaB MGTFs is obviously larger and it increases with increasing of the substrate temperature. Simultaneously, the value of *H*^3^/*E*^2^ is regarded as a ranking index to predict the anti-wear ability of materials [39]. The larger *H*^3^/*E*^2^ value suggests a better wear resistance, which is in line with our nano-scratch results.

## 5. Conclusions

Metallic glass thin films with different compositions, i.e., Cu_50_Zr_50_ (at.%) and Co_56_Ta_35_B_9_ (at.%), were prepared by means of the magnetron sputtering technology. The structure of the MGTFs was effectively tailored via two applied substrate temperatures. The main conclusions are summarized as follows:

1. An enhanced hardness and the elastic modulus are achieved of both MGTFs when the substrate temperature increases from RT to 473 K. The hardness of CuZr MGTFs and CoTaB MGTFs treated at 473 K increased by 13.1% and 9.9%, respectively, and the modulus increased by 12.8% and 1.9% GPa, respectively.
2. Based on the nanoindentation creep tests, compared with the CuZr MGTFs, the CoTaB MGTF deposited at 473 K displays the smaller strain-rate sensitivity exponent, *m*, and a weaker spectrum intensity, suggesting its better creep resistance.
3. The theoretically calculated STZ volume of the CoTaB MGTF is smaller than that of ZrCu MGTF; moreover, it decreases after higher deposited temperature.

According to the nano-scratch analysis, the CoTaB MGTF deposited at 473 K performs a larger *R* value, which reflects the relationship between the penetration depth and the residual depth. Simultaneously, it shows the smaller scratch width and the larger *H*^3^/*E*^2^ value, indicating its better tribological property.

## Figures and Tables

**Table 1:** The values of characteristic depths during nanoindentation tests. e.g., the initial and the maximum displacement of the creep stage, *h*_0_, and *h*_max_, the final deformation depth, *h*_f_, and the deviation between the maximum displacement and the final deformation depth, (*h*_max_ − *h*_f_), of various MGTFs deposited on different substrate temperatures.

Sample	*h*_0_ (nm)	*h*_max_ (nm)	*h*_f_ (nm)	(*h*_max_ − *h*_f_) (nm)
CuZr_RT	243 ± 3	247 ± 2	173 ± 2	74
CuZr_473 K	226 ± 2	229 ± 1	157 ± 3	72
CoTaB_RT	157 ± 1	178 ± 2	113 ± 2	65
CoTaB_473 K	167 ± 2	169 ± 1	108 ± 2	61

**Table 2:** Comparison of the modulus (*E*) and the hardness (*H*) of the Cu_50_Zr_50_ and the Co_56_Ta_35_B_9_ metallic glasses.

Compositions	Cu_50_Zr_50_ *E* (GPa)	Cu_50_Zr_50_ *H* (GPa)	Co_56_Ta_35_B_9_ *E* (GPa)	Co_56_Ta_35_B_9_ *H* (GPa)
BMGs	88 ± 3 [19,22,23,24]	5.5 ± 2.1 [19,22,23,24]	225 ± 10 [20,21]	17.5 ± 1.5 [20,21]
MGTFs in other works	108 ± 4 [19,22,24]	5.8 ± 3.0 [19,22,24]	-	-
MGTFs in this works (RT)	114 ± 1	6.1 ± 0.2	171 ± 1	12 ± 0.2

**Table 3:** The values of fitting parameters obtained by Equation (1) of various MGTFs deposited on different substrate temperatures. *h*_1_ and *h*_1_ are the indentation depths, *τ*_1_ and *τ*_2_ are the characteristic relaxation times for the activation of the *i*-th anelastic process, and *μ*_0_ is a constant proportional to the viscosity coefficient of the last dashpot.

Sample	*h*_1_ (nm)	*τ*_1_ (s)	*h*_2_ (nm)	*τ*_2_ (s)	*μ*_0_ (s/nm)
CuZr_RT	2.95 ± 0.06	4.00 ± 0.06	3.09 ± 0.01	0.290 ± 0.002	27 ± 2.50
CuZr_473 K	2.08 ± 0.05	5.06 ± 0.03	2.75 ± 0.02	0.245 ± 0.001	51 ± 1.04
CoTaB_RT	1.87 ± 0.04	5.41 ± 0.04	1.51 ± 0.01	0.387 ± 0.001	67 ± 0.08
CoTaB_473 K	1.50 ± 0.02	6.85 ± 0.03	1.17 ± 0.01	0.240 ± 0.002	77 ± 2.65

## Data Availability

Data sharing is not applicable to this article.

## Supplementary Materials

The following are available online at https://www.mdpi.com/1996-1944/14/4/999/s1, Figure S1: The first peak of XRD patterns fitted by pseudo-Voigt function of various MGTFs. (a) The CuZr MGTFs prepared at RT. (b) The CuZr MGTFs prepared at 473 K.for CuZr MGTFs prepared at RT and 473K. (c) The CoTaB MGTFs prepared at RT. and (d) The CoTaB MGTFs prepared at 473 K., Figure S2: The diagram of the nano-scratch process, which includes the scratch track before scratch process (BSP), during scratch process (DSP) and after scratch process (ASP).
