# Supplementary material for: Enhanced Mechanical Properties of Metallic Glass Thin Films via Modification of Structural Heterogeneity
Source: Materials (Basel). 2021 Feb 20;14(4):999. doi: 10.3390/ma14040999 (PMC7924064; doi:10.3390/ma14040999)
Supplement: Supplementary file 1 [file materials-14-00999-s001.pdf]

Enhanced mechanical properties of metallic glass thin films via modification of structural heterogeneity.

Xindi Ma [1], Kang Sun [1,*], Peiyou Li [2], Nizhen Zhang [1], Qing Wang [1] and Gang Wang [1,*]

[1] Institute of Materials, Shanghai University, Shanghai 200444, China; xindima@shu.edu.cn (X.M.); dadani@shu.edu.cn (N.Z.); qingwang@shu.edu.cn (Q.W.)
[2] School of Materials Science and Engineering, Shaanxi University of Technology, Hanzhong 723001, China; lipeiyou112@163.com
* Correspondence: sunkang@shu.edu.cn (K.S.); g.wang@shu.edu.cn (W.G.)

I. The analysis of XRD patterns of various MGTFs.

The first peak of XRD patterns can be well fitted by the following pseudo-Voigt function as the solid gray lines shown in Figures. S1(a)–(d). The q value of the CuZr MGTFs moves from 36.66 ± 0.02 and 36.44 ± 0.03 with increasing the substrate temperature from RT to 473 K. The values of q for CoTaB MGTFs prepared at RT and 473 K are 45.05 ± 0.04 to 44.99 ± 0.03 respectively.

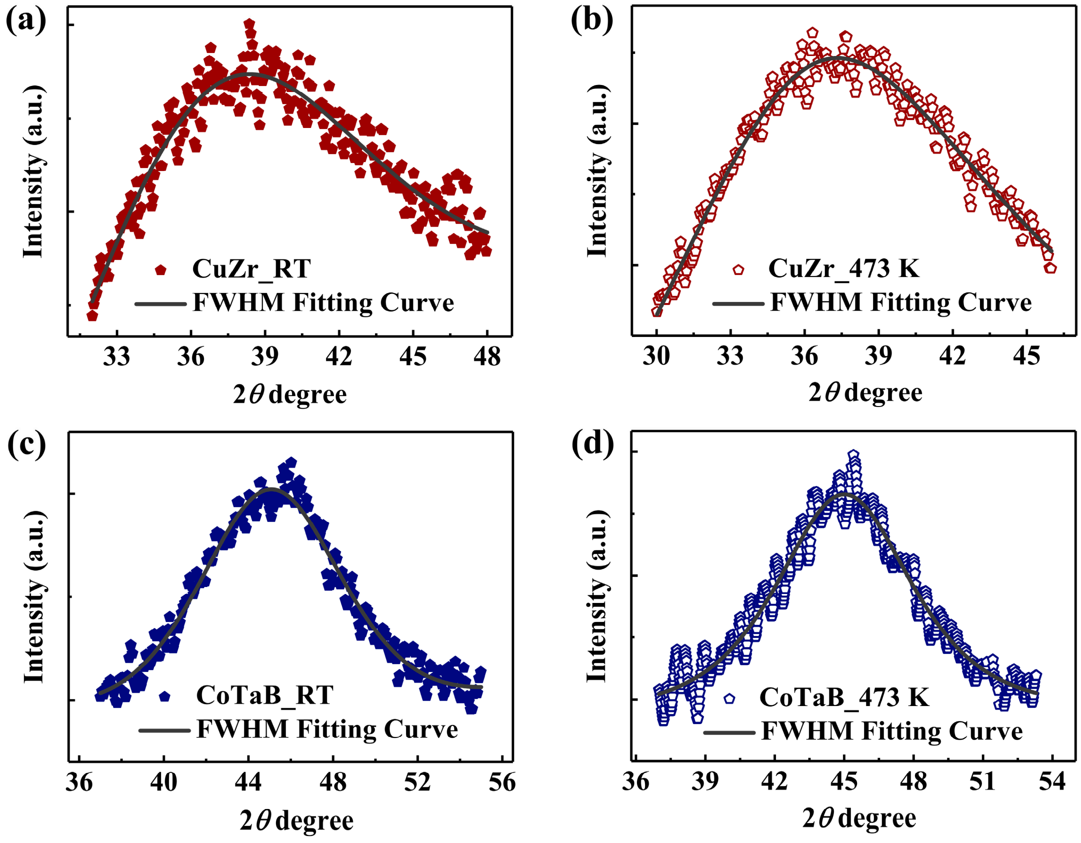

Figure S1. The first peak of XRD patterns fitted by pseudo-Voigt function of various MGTFs. (**a**) The CuZr MGTFs prepared at RT. (**b**) The CuZr MGTFs prepared at 473 K.for CuZr MGTFs prepared at RT and 473K. (**c**) The CoTaB MGTFs prepared at RT. and (**d**) The CoTaB MGTFs prepared at 473 K.

II. The diagram of the nano-scratch process.

Figure S2 shows the representative surface profile of the MGTF scratched with ramping normal loads. The total length of scratches is 200 μm. For each scratch test, three sets of surface profiles along the scratch track were measured, including the track profile before scratch process (BSP), during scratch process (DSP) and after scratch process (ASP). The BSP was obtained by scanning along the prescribed full scratch route prior to the scratch test in order to eliminate the lateral force in the BSP stage. The scratch test was carried out with a ramping load up to 1 mN, and the corresponding DSP was recorded. After the scratch test, the ASP was obtained by scanning along the scratch track again with 0.015 mN normal load. The difference between DSP and BSP indicates the total scratch depth including elastic/plastic deformation and surface damage during the scratch process.

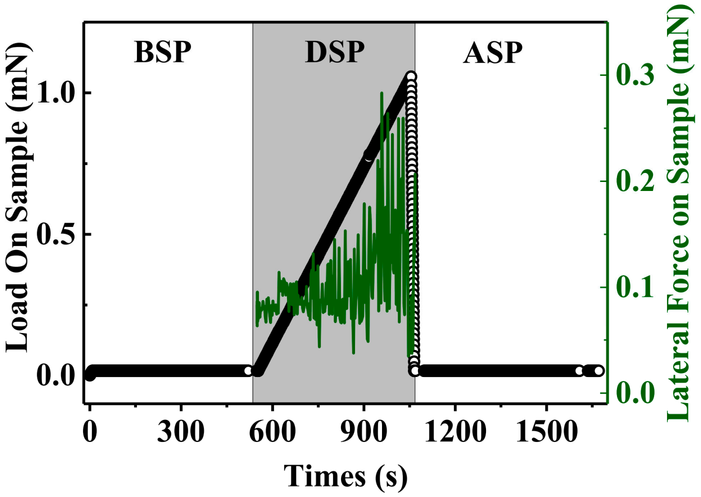

Figure S2. The diagram of the nano-scratch process, which includes the scratch track before scratch process (BSP), during scratch process (DSP) and after scratch process (ASP).
